# Identification of an Ara-C resistance-related gene risk score and the role of S100A4 in AML via NR6A1-dependent activation and p53 regulation

**DOI:** 10.3389/fphar.2025.1574759

**Published:** 2025-06-13

**Authors:** Li Wang, Aoshuang Huang, Bingqing Cheng, Xiuying Hu, Jishi Wang

**Affiliations:** ^1^ Department of Translational Medicine, School of Basic Medical Sciences, Guizhou Medical University, Guiyang, Guizhou, China; ^2^ Department of Hematology, Affiliated Hospital of Guizhou Medical University, Guiyang, Guizhou, China; ^3^ Hematopoietic Stem Cell Transplantation Center of Guizhou Province, Key Laboratory of Hematological Disease Diagnostic & Treat Centre of Guizhou Province, The Affiliated Hospital of Guizhou Medical University, Guiyang, Guizhou, China; ^4^ Department of Internal Medicine, Clinical Medicine College, Guizhou Medical University, Guiyang, Guizhou, China

**Keywords:** S100A4, Ara-C resistance, risk prognostic model, NR6A1, transcription factor, acute myeloid leukemia

## Abstract

**Indroduction:**

Ara‐C (cytarabine) resistance remains a significant contributor to the poor clinical outcomes in adult acute myeloid leukemia (AML). However, predicting Ara‐C resistance and developing effective targeted therapies remain challenging.

**Methods:**

In this study, we integrated transcriptional data from Ara‐C‐resistant cell lines in the GEO database and the TCGA‐LAML cohort to establish an Ara‐C resistancerelated gene risk score (ARRGRS). Kaplan‐Meier survival analysis revealed that AML patients with high ARRGRS had significantly worse prognosis compared to those with low ARRGRS in both cohorts. Additionally, ARRGRS effectively predicted chemotherapy response in AML patients across both cohorts. To further elucidate the mechanisms underlying Ara‐C resistance, we constructed Ara‐C‐resistant AML cell lines and validated our findings using qPCR, Western blotting, flow cytometry (FCM), and in vivo experiments.

**Results:**

We discovered that high expression of S100A4 promotes Ara‐C resistance in AML. Mechanistically, we identified that the transcription factor NR6A1 directly binds to the S100A4 promoter, enhancing its transcriptional activity. Subsequently, S100A4 upregulates p53 expression, thereby promoting AML cell proliferation and resistance to Ara‐C.

**Discussion:**

In summary, our comprehensive investigation of the ARRGRS not only deepens the understanding of Ara‐C resistance mechanisms but also provides promising insights for targeting S100A4 to inhibit tumor growth and overcome chemotherapy resistance in AML.

## Introduction

Acute myeloid leukemia (AML) is a malignant hematological disease that poses significant threats to patients’ lives and imposes a substantial economic burden on society (Carter et al., 2020). Currently, the diagnosis and treatment of AML rely on various methods, including chemotherapy with cytarabine (Ara-C) (Forsberg and Konopleva, 2024). Ara-C is a cytosine nucleoside analog that inhibits cell proliferation by interfering with DNA synthesis, thus exerting an anticancer effect (DiNardo et al., 2020a). Ara-C acts mainly in the S phase of the cell cycle, preventing the elongation of the DNA strand by inhibiting the activity of DNA polymerase (Wang et al., 2022). In recent years, research on Ara-C in the treatment of AML has made significant strides, both in understanding its mechanisms and in improving clinical outcomes. Ara-C remains a cornerstone of AML therapy, often used in combination with anthracyclines in the “7 + 3”regimen, which has been the standard of care for decades (Kantarjian H. et al., 2024). Nevertheless, the emergence of drug resistance presents a formidable obstacle, particularly in the context of long-term cure rates, which are notably less than satisfactory (Kantarjian et al., 2021; Bazinet and Kantarjian, 2023). This challenge is further compounded by the fact that AML cells can develop resistance to Ara-C following extended exposure. This resistance is intricately linked to the regulation of the cell cycle. If AML cells are able to reduce their residence time in S phase or accelerate cell cycle progression by modulating cell cycle-related genes then they could reduce the duration of action of Ara-C, thereby decreasing the killing effect of the drug (Ling et al., 2023). In addition, some studies have found that drug-resistant AML cells may resist the induction of apoptosis by Ara-C by upregulating certain genes associated with cell cycle arrest, such as TP53 (Bruserud et al., 2024, Holtan et al., 2023, Pei et al., 2020, Selheim et al., 2024). Thus, cell cycle regulation plays a key role in the development of cytarabine resistance in AML cells. However, the development of cytarabine resistance remains a major challenge, limiting the efficacy of treatment. Previous studies have identified some genes associated with Ara-C resistance, but the underlying mechanisms are not fully understood.

This study aims to establish a prognostic diagnostic model for acute myeloid leukemia based on Ara-C resistance-related genes and investigate the mechanism by which the key gene S100A4(S100 calcium binding protein A4), activated by the transcription factor NR6A1 (nuclear receptor subfamily 6 group A member 1), mediates Ara-C resistance in acute myeloid leukemia cells through the p53 cell cycle signaling pathway. S100A4, which is also referred to as FSP1, MTS1, or metastasis, is a widely recognized oncoprotein that promotes metastasis and possesses strong tumor-promoting properties (Liu et al., 2021). It is a member of the S100 superfamily of Ca^2+^-binding proteins and is expressed not only by cancer cells but also by a variety of stromal cells (Liu et al., 2019). It has been shown that exosomes secreted by bone marrow mesenchymal stem cells can promote the proliferation, migration and drug resistance of AML cells by up-regulating the expression of S100A4 in AML cells (Lyu et al., 2021). By comparing the nuclear proteome and transcriptome of AML progenitor cells with those of normal human CD34 cells, some researchers have found that knockdown of S100A4 affects the survival of AML cell lines, but not normal hematopoietic stem cell progenitors (Alanazi et al., 2020). This suggests that S100A4 is critical for AML survival and may be a therapeutic target for AML. However, the molecular mechanism by which S100A4 affects AML disease progression and drug resistance has not yet been clarified. NR6A1 also called RTR, NR61, homodimerizes and binds to DNA. Existing studies have reported that NR6A1 is aberrantly expressed in a variety of solid tumors, e.g., hepatocellular carcinoma (Lin et al., 2022), gastric carcinoma (Zhou et al., 2020), and that aberrant expression is closely associated with tumor progression. The DNA methylation indicator of NR6A1 was found to be prognostic in a validation study of potential prognostic DNA methylation biomarkers in patients with acute myeloid leukemia (Šestáková et al., 2022).

The novelty of this study stems from its comprehensive investigation into the role of S100A4 in AML, offering insights that could pave the way for the development of innovative therapeutic strategies targeting this gene. While previous research has highlighted the significance of Ara-C in AML treatment, the emergence of drug resistance poses a persistent challenge. To tackle this issue, we explored the potential Ara-C-resistance mechanisms by culturing Ara-C-resistant cell lines and performing bioinformatics analysis. Molecular subtypes and an Ara-C-resistance-related gene risk score (ARRGRS) were constructed to predict the overall survival of AML patients and to classify AML patients who could benefit from chemotherapy and immune therapy. We further identified S100A4 as the putative gene participating in the process of Ara-C resistance using functional analysis and experimental assays. Specifically, we sought to elucidate the mechanism through which the transcription factor NR6A1 activates the key gene S100A4, which mediates Ara-C resistance in AML cells through the P53 signaling pathway.

## Methods

### Clinical samples

The clinical samples were bone marrow samples collected from patients with AML and normal donors in the Department of Hematology, The Affiliated Hospital of Guizhou Medical University from 2022 to 2024. The patient’s condition was diagnosed using morphological, cytochemical, and immunotyping. Patients with AML were classified as “newly diagnosed” and “relapse” by diagnosis. Therefore, the clinical samples were divided into three groups: “normal donors” (n = 14), “newly diagnosed” (n = 14) and “relapse” (n = 14). Detailed data are shown in Table 1. Written informed consent was obtained from the individuals for the publication of any potentially identifiable images or data included in this article. The clinical samples in this paper were approved by the Ethics Committee of the Affiliated Hospital of Guizhou Medical University for basic research (Approval No. 2023–253).

**TABLE 1:** Correlation between S100A4 expression and clinical parameters of 30 AML patients.

Variabies	Cases	S100A4 expression		P value
		High	Median	
Sex				0.6756
Female	14	9	5	
Male	16		8	
Age				0.4612
<60	15	7	8	
≥60	15	10	5	
WBC(*10^9/L)				0.3328
<10	19	9	10	
≥10	11	8	3	
Blasts in bone marrow (%)				0.7489
<50	39	26	13	
≥50	29	10	19	
FAB classificatopn				0.7754
M0	2	2	0	
M1	7	4	3	
M2	4	2	2	
M4	10	5	5	
M5	7	4	3	
Cytogentics				0.8079
Favorable	10	5	5	
Intermediate	6	4	2	
Unfavorable	14	8	6	
PLT (*10^9/L)				0.8215
<50	12	6	6	
≥50	18	11	7	
CR				0.0580
No	14	11	3	
Yes	16	6	10	

### Establishment of Ara-C-resistant cell lines

The construction of Ara-C-resistant cell lines was validated using IC50. Following a 24-hour exposure to Ara-C, the cell survival was assessed via a CCK-8 assay. The IC50 values for THP-1 (IC50 = 6.34 μM), U937 (IC50 = 13.15 μM), THP-1/R (IC50 = 36.01 μM), and U937/R (IC50 = 90.40 μM) were determined. The IC50 values of THP-1/R and U937/R were considerably higher than those of THP-1 and U937, indicating the success of the Ara-C-resistant strain construction.

### Datastes

AML clinical samples were retrieved from TCGA (https://www.cancer.gov/ccg/research/genome-sequencing/tcga) database as the training set, while data from the Beat AML (https://github.com/radivot/AMLbeatR) database were utilized as the validation set. Ara-C relate different expression genes were downloaded from the Gene Expression Omnibus (GEO: GSE52919) database (https://cancergenome.nih.gov/, accessed on 2 JUN 2024) to function as independent external verification queues.

### Data visualization and differentially expressed genes (DEGs) analysis

The packages in R language mentioned below were employed for data visualization. The differential expression of mRNAs was evaluated using the “Limma” package in R language, with thresholds of p < 0.05 and log2|fold change| >1. The expression levels and distributions of DEGs between C1 and C2 were analyzed using the “Pheatmap” package in the R language.

### Survival analysis

The survival analysis was conducted using the “survival” package in the R language, and the OS of patients belonging to different clusters (C1 and C2) were analyzed and evaluated. Furthermore, a comparison was made between the 1-,3-and 5 years OS of high- and low-risk groups in both the training and validation sets.

### Functional enrichment analysis

GO and KEGG were conducted using the “Cluster Profilter” package in R language, false discovery rate (FDR) < 0.05. The DEGs was subjected to pathway enrichment analysis using GSEA: (https://www.broadinstitute.org/gsea/). Normalize enrichment score (NES): The normalized enrichment score after correction was normalized by the data of the gene set; NOM p-val: The p-value obtained by statistical analysis of ES value represents the reliability of the result; FDR q-val: The p-value after multiple hypothesis testing correction represents the probability of false positive results, The, the smaller the p-value, the more significant. |NES|>1, FDR <0.25, p < 0. 05 was considered statistically significant.

### Identification of Ara-C related prognostic genes

Univariate Cox regression analysis was adopted to obtain Ara-C related genes that exhibited significant associations with OS in AML patients (hazard ratio, HR = 95%, p < 0.05). The “ggforest” package in R language was utilized to construct Figure 3A. LASSO Cox regression analysis was performed on Ara-C related genes to eliminate any false positive Ara-C related genes that may be associated with prognosis. The “glmnet” package in R language was utilized to generate Figure 3 Ara-C related genes were selected according to the minimum λ value for constructing the risk prognostic model.

### Characterization of immune landscape

The CIBERSORT (https://cibersortx.stanford.edu/) in conjunction with the LM22 feature matrix was applied to analyze the differences in immune infiltration of 22 immune cells among different groups. The Pearson product-moment correlation coefficient was utilized to compute the correlation among immune cells, while the Mantel test was employed to statistically analyze the correlation between the risk score matrix and the immune cell matrix. r = 0–1 represents correlation, with higher values indicating stronger correlation, and p < 0.05 was considered statistically significant.

### Construction of the Ara-C related risk prognostic model

The risk scoring formula is as follows: $Risk\;score=\sum_{i=1}^{n}coef_{i}*x_{i}$ . The term “Coefi” denotes the coefficient, while “Xi” represents the normalized count of each core gene. Receiver Operating characteristic (ROC) curve was generated using the R language package “time ROC”. The accuracy of the prognostic model in predicting the 1 -, 3 - and 5-year OS of AML patients was assessed by calculating the Area Under Curve (AUC) in both the training and validation datasets.

### Extraction of bone marrow mononuclear cells from clinical samples

5 mL of bone marrow from AML patients or normal donors was collected by bone marrow puncture under routine sterile conditions and preserved with EDTA anticoagulation. Bone marrow was diluted 1:1 in equal volume with saline and was slowly added along the wall to a centrifuge tube pre-loaded with Ficoll (Solarbio Technologies, Beijing, China) separation solution, and Ficoll was 1:1 with diluted bone marrow. After centrifugation at 2,000 rpm for 15 min at room temperature, the intermediate white cell layer was aspirated and transferred to a new centrifuge tube. After centrifugation at 1,500 rpm for 5 min, the supernatant was discarded. The remaining precipitate, namely, bone marrow mononuclear cells, was retained after being washed three times with saline and subsequent discarding of the supernatant.

### Cell culture

The authenticity of THP-1 and U937 human leukemia cell lines was confirmed by STR analysis, and they were cultured in a 5% CO_2_ incubator at 37°C using RPMI1640 medium containing 10% fetal bovine serum. Drug-resistant variants, namely, THP1R and U937R, were generated by supplementing 1% penicillin (100 units/mL) and streptomycin (100 mg/mL) to the medium along with increasing concentrations of Ara-C. The drug concentration was gradually escalated, repeating this process three to five times at each concentration after the cells have proliferated to a normal shape. The drug induction was maintained for a duration of 6–8 months until the cells achieved a stable state at the final concentration.

### Real-time PCR

The extraction of total RNAs from cells was performed using Trizol reagent (Invitrogen, Carlsbad, CA, United States). The mixture was vigorously shaken for 15 s after the addition of chloroform, followed by incubation at room temperature for 3 min. The samples were centrifuged at 12,000 rpm for 15 min at 4°C, and the supernatant was retained. An equal volume of isopropanol was added, followed by centrifugation at 12,000 rpm for 10 min at 4°C. Subsequently, the supernatant was discarded. The RNA precipitate was washed with 500 μL of 75% ethanol. Subsequently, the samples were subjected to centrifugation at a speed of 7,500 rpm for a duration of 3 min at a temperature of 4°C, followed by removal of the supernatant. The samples were air-dried for 5 min at room temperature to allow ethanol evaporation, followed by addition of DEPC water and measurement of concentration. cDNA was extracted using a reverse transcription kit (MedChemExpressMCE, United States of America). Real-time PCR was performed using SYBR Green PCR Master Mix (MedChemExpressMCE, United States) kit and PRISM 7500 realtime PCR Detection System (Thermo Fisher Scientific, United States). Relative expression of the target genes was calculated using β-actin as the reference through comparative cycle threshold (CT) values (2^−ΔΔCT^). The following human primers were used in Supplementary Table S1.

### Western blotting

The primary antibodies against S100A4 and NR6A1 (Affinity Biosciences, United States of America) were diluted at 1:10,000 and 1: 10,00, cyclin D1 (1:30,000, Proteintech, China), CDK4 (1:4,500, Proteintech, China), p53 (1:20,000, Proteintech, China), respectively, the β-actin primary antibody (Wuhan Sanying, China) was diluted at 1:3,000. The HRP-conjugated secondary antibody was diluted to 1:10,000 (Proteintech, China). Protein lysates were extracted from cells by adding 1 mM PMSF to the RIPA lysis buffer (Solarbio Science and Technology). The mixture was vigorously shaken and incubated on ice for 30 min. Subsequently, the supernatant was obtained by centrifugation at 12,000 rpm for 15 min at 4°C. The concentration of protein was determined using the BCA Protein Assay kit (Pierce, Hercules, CA, United States). The proteins were mixed with Loading buffer 1:4 and boiled at 100°C for 10 min 40 μg of proteins were then added to a 10% SDS-PAGE gel and electrophoresed into the separation gel at a constant voltage of 80 V followed by switching to a stable voltage of 120 V. At the end of electrophoresis, the separated proteins were transferred onto PVDF membranes and rotated at 250 mA for 1 h. After shaking with PBS containing 5% skim milk on a shaker for 2 h at room temperature, the membranes were washed. The primary antibodies were then incubated for more than 8 h at 4°C. After washing the membranes, secondary antibodies were incubated for at room temperature for 45 min. All protein bands were visualized using the Enhanced Chemistry kit (7Sea Biotech, Shanghai, China). β-actin was used as the internal reference.

### Cell counting Kit-8 assay (CCK8)

CCK8 assay was used to detect the sensitivity of leukemia cell lines to Ara-C. The cells were inoculated into individual wells of a 96-well plate at a seeding density of 3 ×10^4^ cells/100 μL, with five replicates per experimental group. After subjecting the cells to various concentrations of Ara-c for a duration of 24 h, a volume of 10 μL CCK8 reagent was added into each well, the concentrations of Ara-c in U937 and U937R cell lines were 4, 16, 64, 192, 386, 578, 768 and 1,536 μM, meanwhile the concentrations of Ara-c inTHP-1 and THP-1R cell lines were 0.5, 4, 64, 192, 386, 578, 768 and1536 μM. The absorbance at 450 nm was quantified using a microplate spectrophotometer after co-culturing for 1–2 h. The IC50 value was determined by employing the GraphPad Prism 9.4 software.

### Cell transfection and lentivirus infection

We obtained human S100A4-silencing RNA (sh-S100A4), S100A4-overexpressing lentiviral particles (LV-S100A4), and NR6A1-overexpressing lentiviral particles (NR6A1-OE) from Genechem (Shanghai, China). The controls consisted of cells transfected with an empty vector (EV).

### Detection of viable cells by EDU staining

Cell viability was assessed using the EDU (5-ethynyl-2′-deoxyuridine) incorporation assay. Cells were incubated with 10 μM EDU for 2 h, fixed with 4% paraformaldehyde, and permeabilized with 0.3% Triton X-100. EDU -positive cells were labeled with Alexa Fluor^®^ 488 azide using the Click-iT^®^ EDU Imaging Kit (Thermo Fisher Scientific) and counterstained with Hoechst 33,342. Fluorescence images were captured using a fluorescence microscope, and the percentage of viable cells was calculated by counting EdU-positive cells relative to total nuclei.

### Apoptosis assay

After harvesting and PBS-washing, the cells were subjected to Annexin-V/propidium iodide (PI) staining to assay the apoptotic ratio as per the advised protocol (7Sea Pharmatech, Shanghai, China), followed by flow cytometry (BD Biosciences, San Jose, United States).

### Immunohistochemical (IHC) staining

IHC staining with antibodies against S100A4 were performed to detect protein expression levels following standard operating procedures. Paraffin-embedded tissue sections (4 μm) were dewaxed in an eco-friendly dewaxing agent (three changes, 10 min each), rehydrated through graded ethanol series (100%, 95%, 80%, 5 min each), and rinsed in distilled water. Antigen retrieval was performed using citrate buffer (pH 6.0) under optimized conditions to prevent tissue drying. After cooling to room temperature, sections were washed in PBS (pH 7.4, 3 × 5 min on a shaker). Endogenous peroxidase activity was blocked with 3% H_2_O_2_ (25 min, room temperature, dark), followed by PBS washes. To reduce nonspecific binding, sections were incubated with 3% bovine serum albumin (BSA) or species-matched serum (30 min, room temperature). Primary antibody against the target protein (diluted in PBS) was applied overnight at 4°C in a humidified chamber. After PBS washes, HRP-conjugated secondary antibody (species-matched) was added for 50 min at room temperature. Signal visualization was achieved using DAB chromogen under microscopic monitoring, followed by rinsing in tap water to terminate the reaction. Nuclei were counterstained with hematoxylin (3 min), differentiated in acid ethanol, blued in ammonia water, and rinsed. Sections were dehydrated through graded alcohols (75%, 85%, 100%, 5 min each), cleared in xylene, and mounted with resin. Protein expression was evaluated using bright-field microscopy.

### Xenografted tumor model

NOD-SCID mice were purchased from Model Organisms Center (Shanghai, China). Stably transfected S100A4 cells were resuspended in PBS at a concentration of 5 × 10^6^ cells/100 μL and then subcutaneously injected into the 5-week-old female mice. The mice were randomly divided into four groups: EV1, LV-S100A4, EV2 and sh-S100A4. Once tumors were visible or palpable, mice were treated with Ara-C (60 mg/kg/day for 5 days) by intraperitoneal injection 48 h. Mice were placed on the platform of BLT *In-Vivo* Imaging System (BLT Photon Tech., Guangzhou, China). Tumor weight and diameter were measured every week. All experiments on mice were approved by the Institutional Animal Care and Use Committee of Guizhou Medical University, China. Although there is no blinding in this experiment, it avoids introducing bias in the evaluation of experimental data.

### Statistical analysis

All the images were digitally processed and labeled using ImageJ and Illustrator 2021. Statistical analyses were performed using GraphPad Prism 9 software. Statistical methods covering: T-test (satisfies normal + homogeneous variance), Welch t' test (satisfying normal + not satisfying variance homogeneity), Wilcoxon rank sum test and one-way ANOVA. The value of p < 0.05, 0.01, and 0.001 was set for the thresholds of statistical significance. Data are shown as the mean ± SEM.

## Results

### Functional characterization of Ara-C-resistant AML cell lines

**FIGURE 1:**
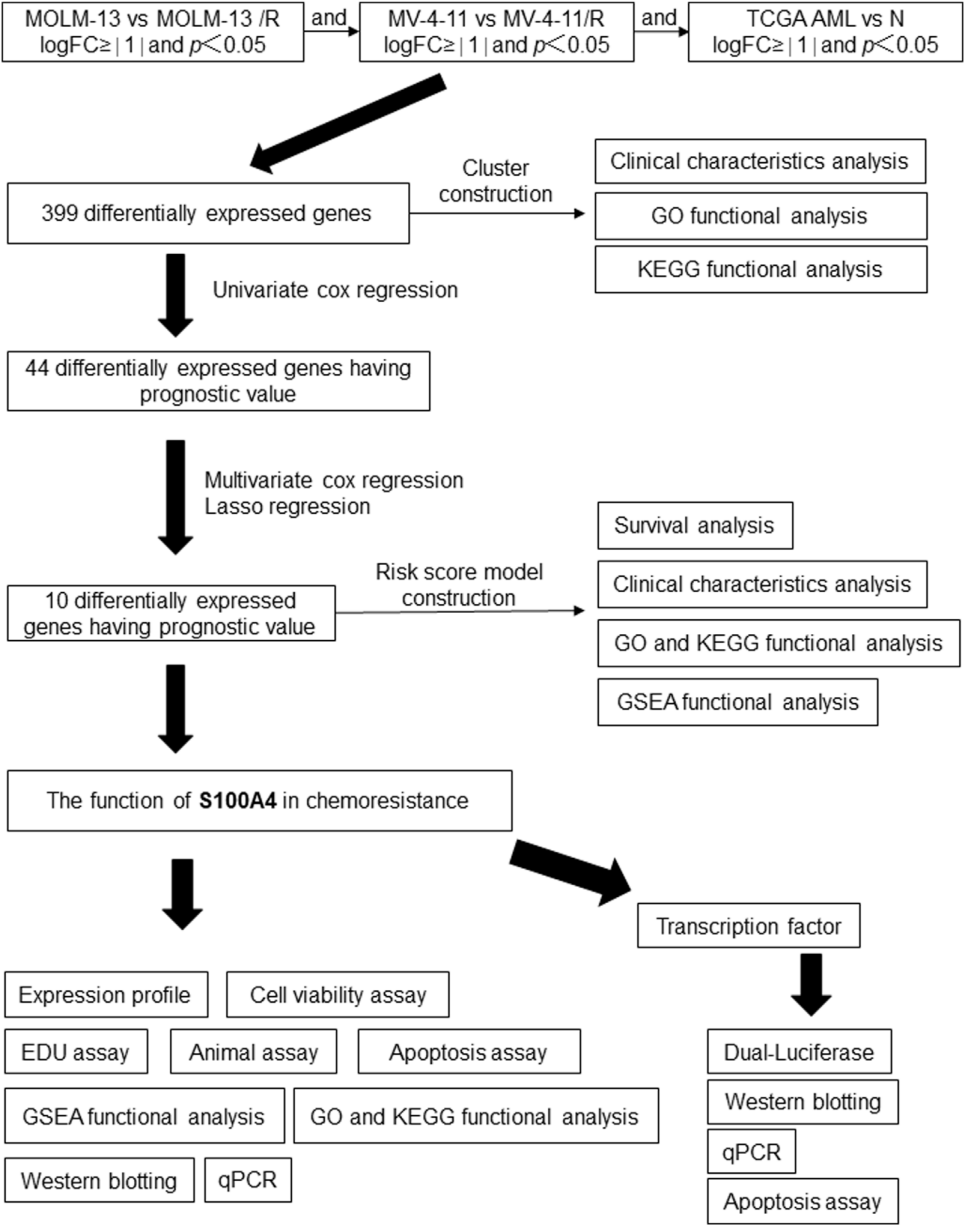
Workflow of the study design.

**FIGURE 2:**
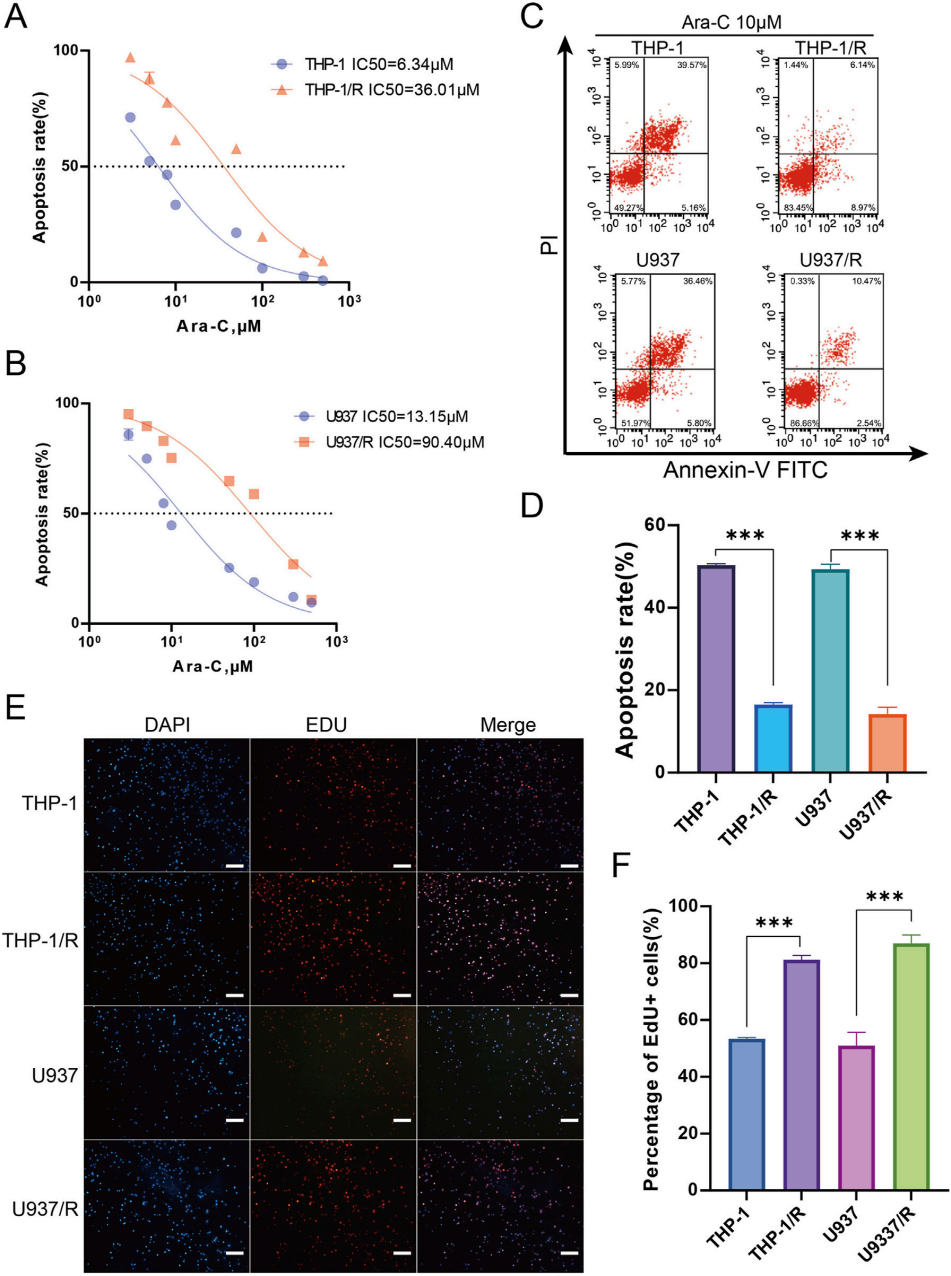
Establishing Ara-C-resistant Cell Lines **(A,B)** Cell viability assay was applied to confirm the establishment of the Ara-C-resistant cell lines. **(C,D)** Apoptosis assay was applied to confirm the establishment of the Ara-C-resistant cell lines. 10 μM Ara-C was used for 24 h for the further experiments. **(E,F)** EDU staining for assessing cell viability. Again, 10 μM Ara-C was used for 24 h for the further experiments. Scale bars, 100 μm. Data are shown as mean ± SD representing three biological replicates. *p < 0.05, **p < 0.01, ***p < 0.001.

In order to explore the potential mechanisms by which AML develops an Ara-C chemotherapy, a research protocol was designed.The protocol is outlined in Figure 1. After we established the stable Ara-C resistant THP-1 cell line (THP-1/R) and U937 cell line (U937/R), the growth inhibitory effects of Ara-C on THP-1, THP-1/R, U937 and U937/R cells were determined by the CCK-8 assay (Figures 2A,B). Following the stimulation of Ara-C, apoptosis in different cell lines, the apoptosis rate was detected by FCM, and the apoptosis rates of THP-1/R and U937/R were smaller than those of THP-1 and U937 (Figures 2C,D). Following the incorporation of Ara-C into the four distinct cell groups, the EDU method was employed to detect live cells. The results demonstrated that the proportion of live cells to total cells was higher in the THP-1/R and U937/R groups compared to the THP-1 and U937 groups (Figures 2E,F). In comparison with control cell lines, THP-1/R and U937/R cell lines exhibited higher IC50 values under Ara-C treatment, a greater number of live cells detected by EDU, and a lower rate of apoptosis.

### Construction of an AML risk prognostic model using Ara-C resistant-related genes

**FIGURE 3:**
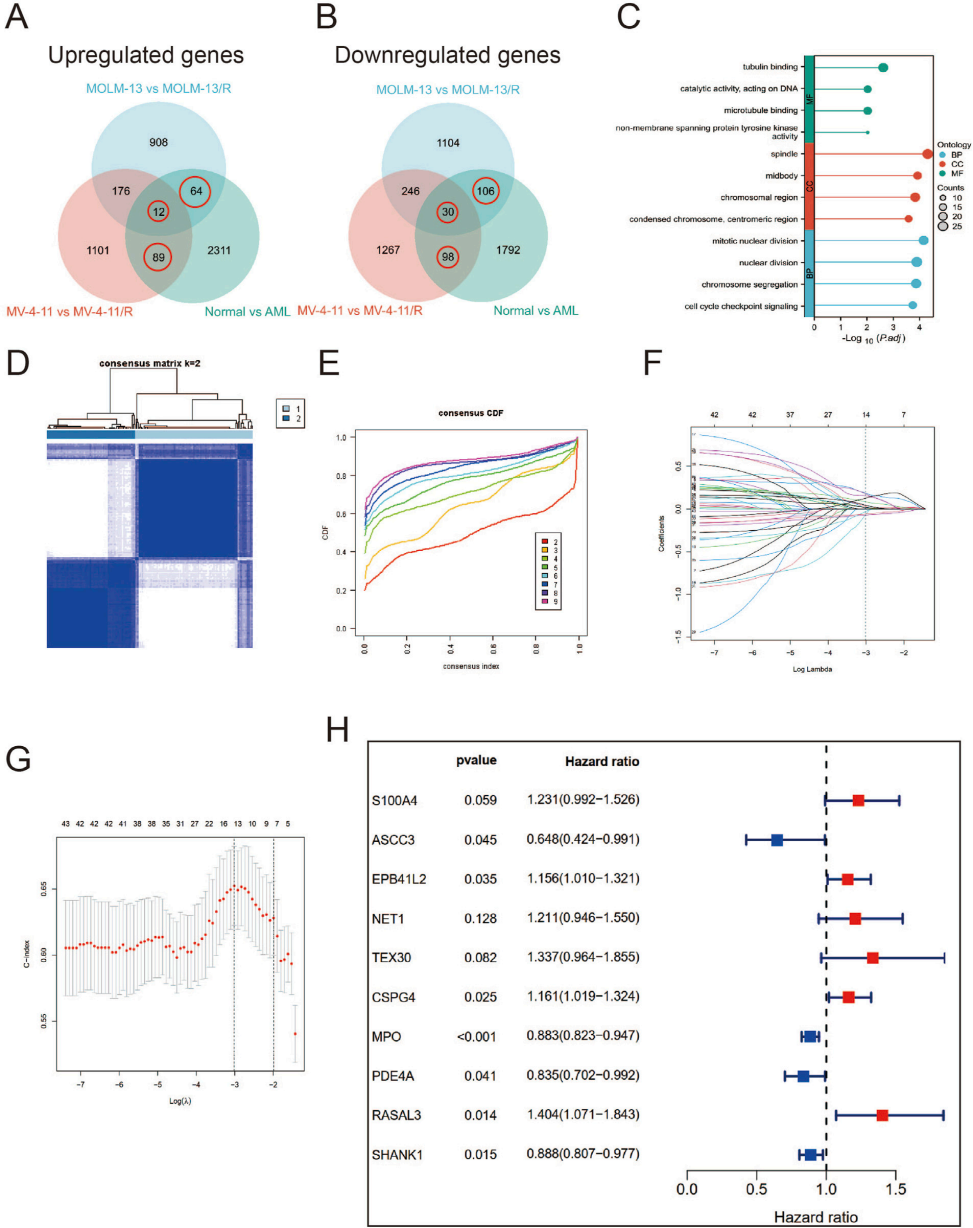
Identifying Hub Genes of Ara-C Resistance and Cluster Analysis; Establishing a Prognostic Risk Score Model. **(A,B)** Ara-C-resistant hub genes were identified. The criterion (|log2 FC| > 1 and adjusted p-value <0.05) was used. *** means p < 0.001. **(C)** GO/KEGG analysis of differential expression genes. **(A)** Consensus clustering heat map was constructed with k = 2. **(D)** Heatmap of consensus clustering matrix when K = 2. **(E)** CDF curve and Delta area curve for K = 2–9. **(F)** LASSO coefficients of four prognostic Ara-C -resistance-related genes in the TCGA-LAML cohort. **(G)** Turning parameter selection via minimum criteria were used for cross-verification in the LASSO regression model. **(H)** The forest plot displays the hazard ratios (HR) and 95% confidence intervals (CI) for each variable included in the multivariate Cox regression model.

Furthermore, we sought to identify differentially expressed genes (DEGs) between Ara-C-resistant and sensitive cell lines (GSE125403). Subsequently, we integrated the DEGs from AML tumor samples (TCGA database) and normal control samples (GTEx database), which resulted in the identification of 399 Ara-C resistance-related genes. These genes were presented as Venn diagrams (Figures 3A,B). These differential genes were then subjected to Gene Ontology (GO) and Kyoto Encyclopedia of Genes and Genomes (KEGG) analysis (Figure 3C). The results demonstrated that these differential genes were predominantly associated with tublin binding, catalytic activity, chromosomal region and segregation, and cell cycle checkpoint signaling. Subsequent cluster analysis of AML patients based on the Ara-C resistance-related genes obtained after differential analysis showed that AML patients could be successfully classified into two clusters (Figures 3D,E). To further investigate the value of Ara-C resistance-related genes in assessing the clinical prognosis of AML, we constructed a risk-prognostic model for AML based on Ara-C resistance-related genes. Initially, univariate Cox regression analysis was conducted to identify 44 genes from the 357 Ara-C resistance-associated genes that exhibited a strong association with AML prognosis (HR = 95%, P < 0.05). To further refine the analysis and eliminate the possibility of false-positive prognostic factors, 10 Ara-C resistance genes were subjected to LASSO Cox regression analysis, which identified those genes that exhibited a high degree of association with AML prognosis (HR = 95%, P < 0.05) (Figures 3F,G) (Tibshirani, 1996). The patients belonging to the AML-TCGA cohort were assigned to high or low ARRGRS groups referring to the median ARRGRS. The expression profiles of 10 prognostic hub genes including S100A4, ASCC3, EPB41L2, NET1, TEX30, CSPG4, MPO, PDE4A, RASAL3 and SHANK1 in the high/low ARRGRS groups were exhibited in the style of a heat map (Figure 3H). In addition, the results of the validation of the proportional risk hypothesis for the 10 genes in the ARRGRS score showed that the individual test p-value of the individual Schoenfeld test for the genes was greater than 0.05, except for the EPB4IL2 gene, for which the p-value of the test was close to the significance level (0.0289), and the p-value of the global Schoenfeld test was 0.2784, and the proportional risk hypothesis was not rejected as a whole, i.e., The covariates in the overall model as a whole satisfy the proportional risk assumption (Supplementary Figure S1).

### Verification of the predictive power of the Ara-C-related gene risk prognostic model and association of the risk prognostic model with TIME of AML

**FIGURE 4:**
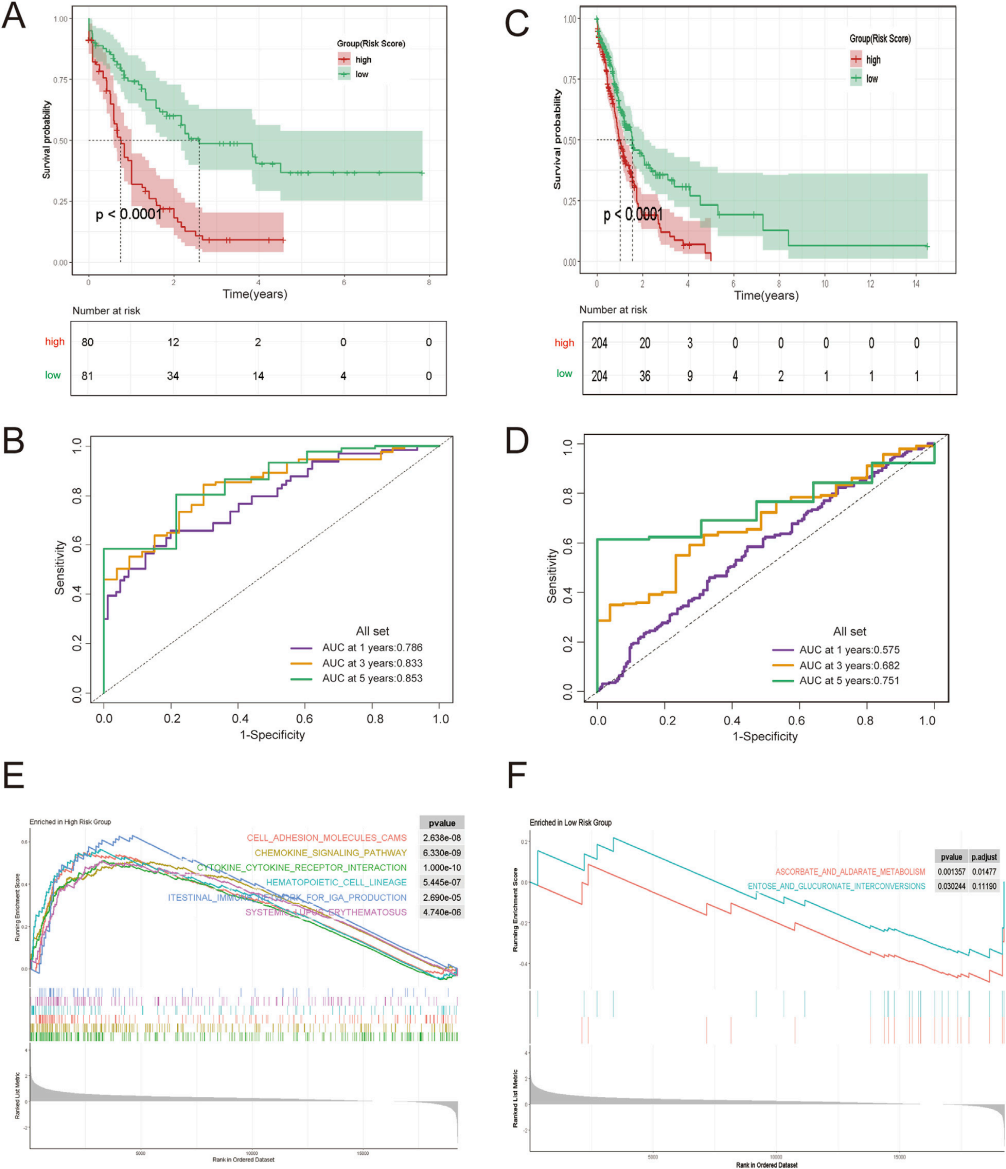
Construction and Validation of an AML Risk Prognostic Model Based on Ara-C-Resistance-Related Genes. **(A)** KM survival curves in the high-ARRGRS and low- ARRGRS risk groups. **(B)** ROC curves of the risk prognostic model predicting the prognosis of AML patients. **(C,D)** Validation of the risk prognostic models in the validation set. **(E)** GSEA analysis of differentially expressed genes in high ARRGRS groups. **(F)** GSEA analysis of differentially expressed genes in low ARRGRS groups. ARRGRS: Ara-C-resistance-related gene risk score.

Next, the correlation between ARRGRS and prognosis was also analyzed. As shown in Figure 4A, the patients belonging to the high ARRGRS groups exhibited a poorer prognosis (p < 0.0001). To verify the predictive value of ARRGRS, ROC curves were drawn. The area under the curves (AUCs) were 0.786, 0.833 and 0.853, respectively, for 1-year, 3-year and 5-year survival (Figure 4B), indicating that ARRGRS could favorably predict the overall survival status of AML patients. The external validation cohort, the Beat AML data base, also confirmed that the ARRGRS groups were occupied with satisfactory overall survival prediction ability (p < 0.0001) (Figure 4C). The area under the curves (AUCs) were 0.575, 0.682 and0.751, respectively, for 1-year, 3-year and 5-year survival (Figure 4D). Next, we explored the potential mechanism of Ara-C resistance by performing GSEA. The results of the GESA using DEGs in the high ARRGRS groups showed that the process of cell adhesion, chemokine signaling pathway, cytokine-cytokine receptor interaction, hematopoietic cell lineage and intestinal immune network for IgA production were involved in the development of Ara-C resistance (Figure 4E). The results of the GESA using DEGs in the low ARRGRS groups showed that the ascorbate metabolism and glucuronate interconversions were involved in the development of Ara-C resistance (Figure 4F).

### S100A4 were closely associated with poor prognosis of AML

**FIGURE 5:**
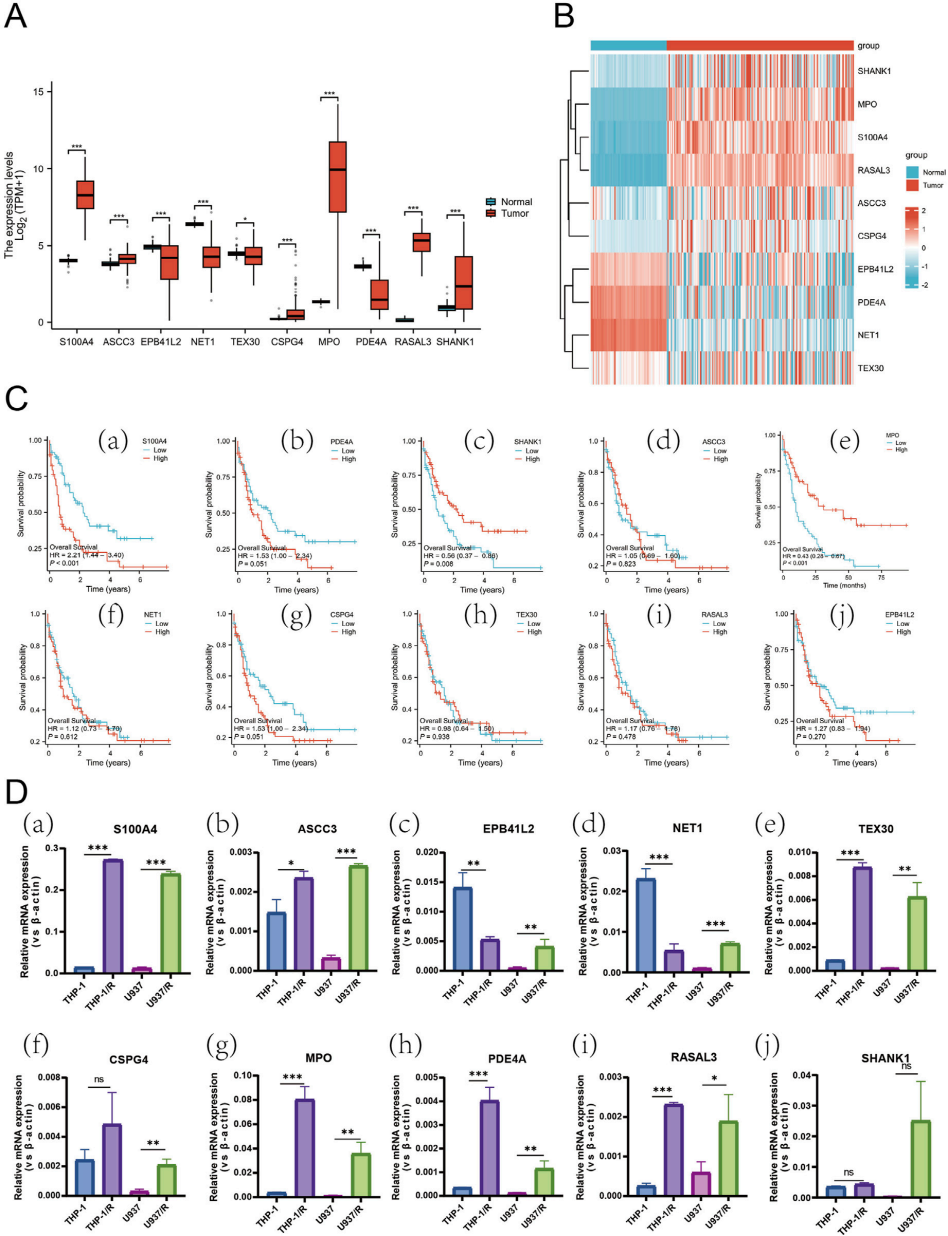
Expression Profiles of Hub Genes in AML and Differential mRNA Levels Across Cell Lines. **(A)** Box plots showing differences in the expression of the 10 Hub-genes in normal and AML patient samples. **(B)** Heatmap of the DEGs expression levels in the normal and AML patient groups. **(C)** The relationship of S100A4 (a), PDE4A (b), SHANK1 (c), ASCC3 (d), MPO (e), NET1 (f), CSPG4 (g), TEX30 (h), RASAL3 (i) and EPB41L2 (j) with OS in AML patients in TCGA database. **(D)** the mRNA expression level of S100A4 (a), ASCC3 (b), EPB41L2 (c), NET1 (d), TEX30 (e), CSPG4 (f), MPO (g), PDE4A (h), RASAL3 (i) and SHANK1 (j) in sensitive and Ara-C-resistant cell lines. Data are shown as mean ± SD representing three biological replicates. *p < 0.05, **p < 0.01, ***p < 0.001, ns, no significant difference.

In order to thoroughly examine the mechanism of Ara-C resistance generation in AML cells, an analysis was conducted of the expression differences of ten genes in normal and AML patients (Figure 5A). S100A4, ASCC3, CSPG4, MPO, RASAL3, and SHANK1 exhibited significantly higher levels of expression in AML patients compared to normal subjects. Conversely, EPB41L2, NET1, TEX30, and PDE4A exhibited higher expression levels in normal subjects and lower levels in AML patients. Gene co-expression heatmap results showed that MPO, S100A4, and RASAL3 were generally lowly expressed in normal subjects and highly expressed in AML patients, while EPB41L2, PDE4A, and NET1 were generally highly expressed in normal subjects and lowly expressed in AML patients (Figure 5B). These findings suggest that these genes associated with resistance to treatment may exhibit differential expression between normal subjects and AML patients. To identify additional genes that are clinically significant, we performed survival analysis on these 10 genes (Figure 5C). The finding that the survival rate is lower in the S100A4 high expression group compared to the low expression group is consistent with our hypothesis that S100A4 overexpression is associated with poorer prognosis. (Figure 5C a). Similarly, the survival rate was lower in the SHANK1 low-expression group compared to the high-expression group (Figure 5c). Additionally, the survival rate was lower in the MPO low-expression group compared to the high-expression group (Figure 5e). This result provides further evidence to support our research hypothesis and suggests that S100A4 may be a potential biomarker for predicting survival outcomes.

Subsequently, an experimental investigation was conducted to ascertain whether there was a discrepancy in the expression of the 10 genes in the AML-sensitive (THP-1, U937) and Ara-C-resistant (THP-1/R, U937/R) cell lines (Figure 5D). RT-PCR results indicated that S100A4, ASCC3, TEX30, MPO, PDE4A, and RASAL3 were expressed in both THP-1 and U937. The expression of S100A4 exhibited the most significant discrepancy, with EPB41L2 and NET1 being elevated in THP-1/R compared to THP-1, yet diminished in U937/R compared to U937.

A comprehensive examination of the expression differences, survival analysis, and the results of the PCR assay screening was conducted. This analysis led to the conclusion that S100A4 has a significant potential to be associated with the development of Ara-C resistance in AML cells.

### S100A4 expression: impact on Ara-C resistance in AML cells

**FIGURE 6:**
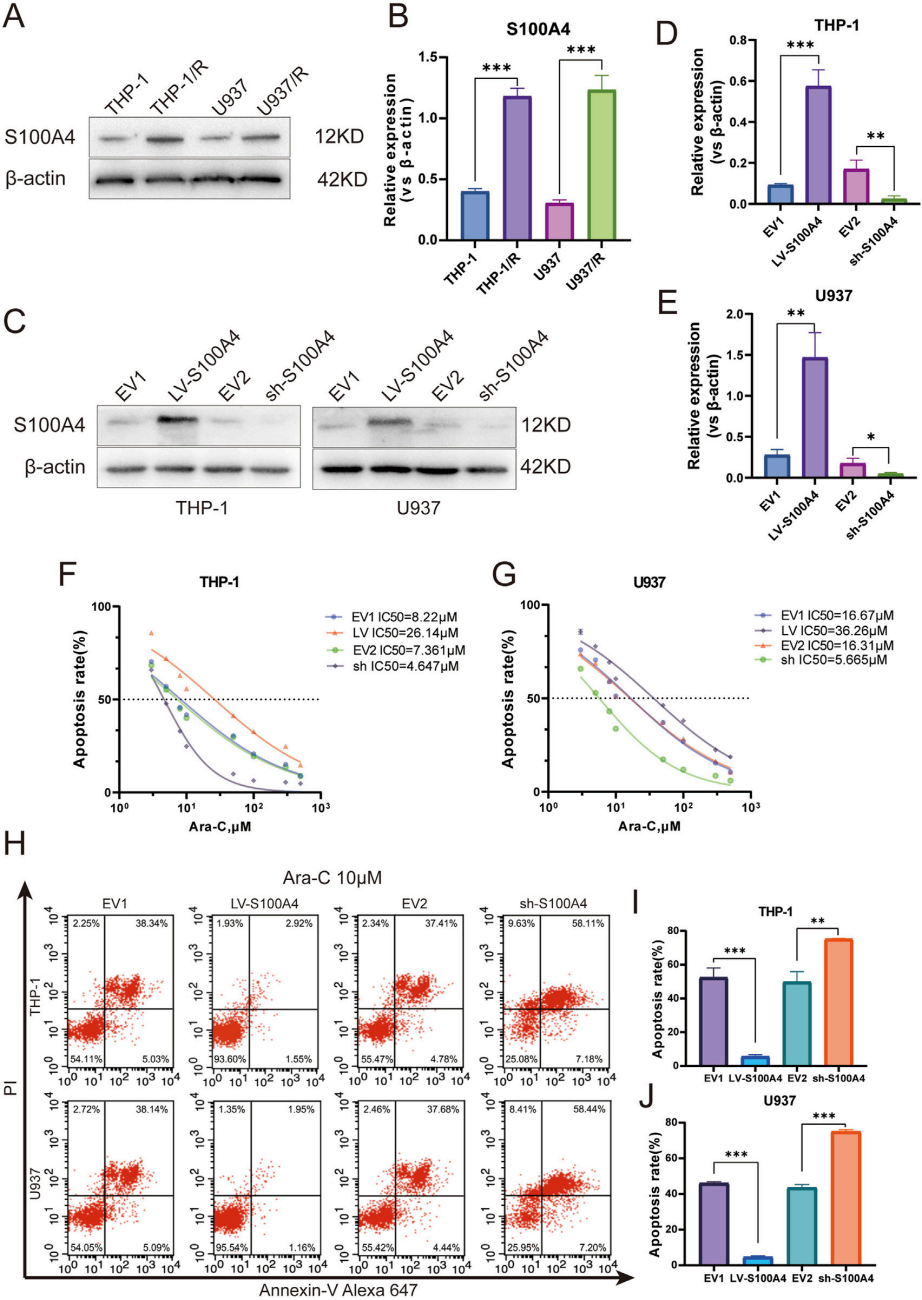
Expression Profile and Functional Analysis of S100A4 in Ara-C-Resistant Cell Lines. **(A)** S100A4 was highly expressed in Ara-C resistant cell lines confirmed by Western blotting. **(B)** The relative gray values were shown in histogram. **(C)** S100A4 was overexpressed or silenced in THP-1 and U937 cell lines determined by western blotting analyses. **(D,E)** The relative gray values were shown in histogram. **(F,G)** Dose-response curves of Ara-C of LV-S100A4 and sh-S100A4 in THP-1 and U937. CCK-8 assay after 24 h of drug exposure determined Cell viability. **(H–J)** The percentage of apoptotic cells was demonstrated by flow cytometry in both cell lines following the overexpressed or silenced of S100A4. Data are shown as mean ± SD representing three biological replicates. *p < 0.05, **p < 0.01, ***p < 0.001, ns, no significant difference.

An examination of S100A4 protein expression in four distinct cell lines revealed that S100A4 expression levels were significantly higher in THP-1/R and U937/R cells compared to THP-1 and U937 cells (Figure 6A). To further explore the role played by S100A4 in the development of Ara-C resistance in AML cells, we used lentivirus to upregulate and downregulate the expression of S100A4 in THP-1 and U937 cell lines, respectively. Western blot verified the regulatory effect at the protein level (Figure 6B), and RT-PCR verified the regulatory effect at the transcriptional level (Figures 6C–E). Following the regulation of S100A4 gene expression in the THP-1 cell line, the median inhibitory concentration (IC50) value of cells exhibiting elevated S100A4 expression increased, while the IC50 value of cells with reduced S100A4 expression decreased (LV-S100A4:IC50 = 26.14 μM; EV1:IC50 = 8.22 μM; sh-S100A4:IC50 = 4.647 μM; sh-S100A4. (EV2:IC50 = 7.361 μM) (Figure 6F). A similar trend was observed in the U937 cell line (LV-S100A4:IC50 = 36.26 μM; EV1:IC50 = 16.67 μM; sh-S100A4:IC50 = 5.665 μM; EV2:IC50 = 16.31 μM) (Figure 6G). Subsequently, we employed FCM to assess the apoptosis rate in different groups following Ara-C (10 μM) stimulation (Figure 6H). The results demonstrated that the lowest apoptosis rate was observed in the LV-S100A4 group, which exhibited high S100A4 expression, and the highest rate was observed in the sh-S100A4 group, which exhibited low S100A4 protein expression in both THP-1 and U937 cells (Figures 6I,J).

Furthermore, we observed that S100A4 expression was significantly elevated in Ara-C-resistant strains. The upregulation of S100A4 expression in sensitive strains demonstrated a modest enhancement in cell resistance to Ara-C, while the downregulation of S100A4 expression exhibited a corresponding increase in cell sensitivity to Ara-C. These findings collectively suggest the potential for S100A4 to play a significant role in the resistance of AML cells to the lethal effects of Ara-C.

### *In vivo* S100A4 overexpression promotes AML tumor cell growth and impairs the therapeutic effect of Ara-C

**FIGURE 7:**
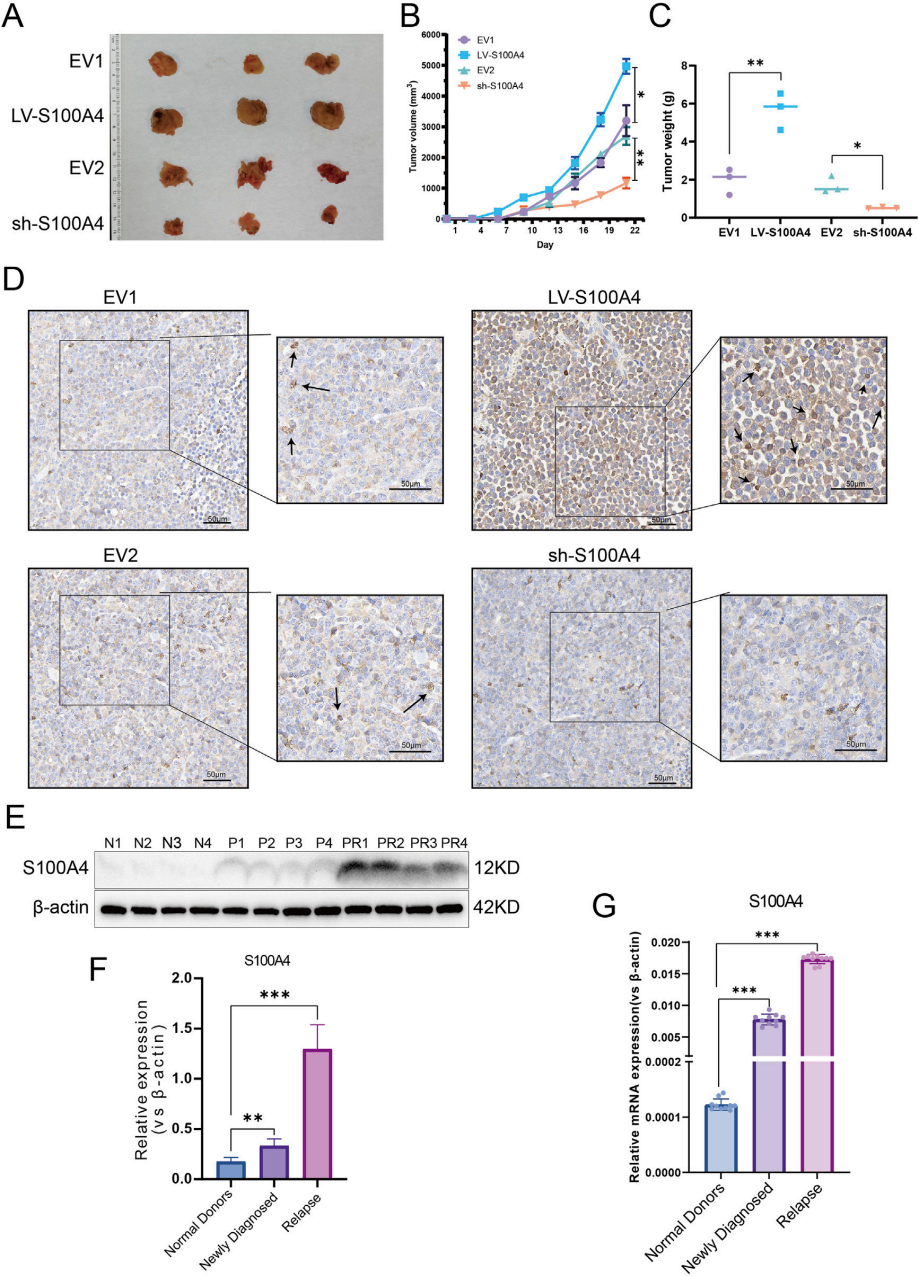
Expression Profile and Functional Analysis of S100A4 *in Vivo* and Association of S100A4 with the Poor AML Prognosis. **(A)** The general view of tumors (EV1, LV-S100A4, EV2 and sh-S100A4 groups). **(B)** Tumor volume curves. **(C)** The tumor weights of EV1, LV-S100A4, EV2 and sh-S100A4 groups. **(D)** The expression of S100A4 was examined in xenograft tumor tissue sections using immunohistochemistry. Scale bars: 50 μm. **(E,F)** The protein expression of S100A4 in the “normal donors group” (n = 4), “newly diagnosed group” (n = 4) and “relapse group” (n = 4). Grey values of S100A4. **(G)** The mRNA expression of S100A4 in the “normal donors group” (n = 10), “newly diagnosed group” (n = 10) and “relapse group” (n = 10). Data are shown as mean ± SD representing three biological replicates. *p < 0.05, **p < 0.01, ***p < 0.001, ns, no significant difference.

Four distinct groups of EV1, LV-S100A4, EV2, and sh-S100A4 cells were injected subcutaneously into NOD/SCID mice, with the administration of Ara-C via the tail vein on the seventh day. The subcutaneous tumor results demonstrated that the LV-S100A4 group, which exhibited high S100A4 protein expression, exhibited the largest tumor volume (Figure 7A), the fastest growth rate (Figure 7B), and the heaviest weight (Figure 7C). Conversely, tumors in the sh-S100A4 group, which exhibited low expression of S100A4 protein, demonstrated the smallest volume, the slowest growth rate, and the lightest weight. The results of IHC analysis after paraffin sectioning of subcutaneous tumors showed that in the *in vivo* model the LV-S100A4 group had the highest expression level of S100A4 protein and the sh-S100A4 group had the lowest expression level (Figure 7D). The *in vivo* experiments demonstrated that AML cells with high S100A4 protein expression exhibited increased resistance to Ara-C resistance, and the inhibition of S100A4 protein expression enhanced the killing effect of Ara-C on AML tumor cells. In addition, our center collected human bone marrow blood samples for testing and found that S100A4 protein expression was higher in patients with primary diagnosed AML than in normal donors, and S100A4 protein levels were higher in patients with relapsed AML than in patients with primary diagnosis and in normal donors (Table 1; Figures 7E,F). The PCR results also showed that relapsed patients had the highest levels of S100A4, and that the group of patients with primary diagnosis AML was higher than the group of donors on average (Figure 7G).

### Activation of S100A4 by the transcription factor NR6A1 in AML increases tumor cell drug resistance through the p53/cyclin-D1 signaling pathway after cell cycle inhibition.

**FIGURE 8:**
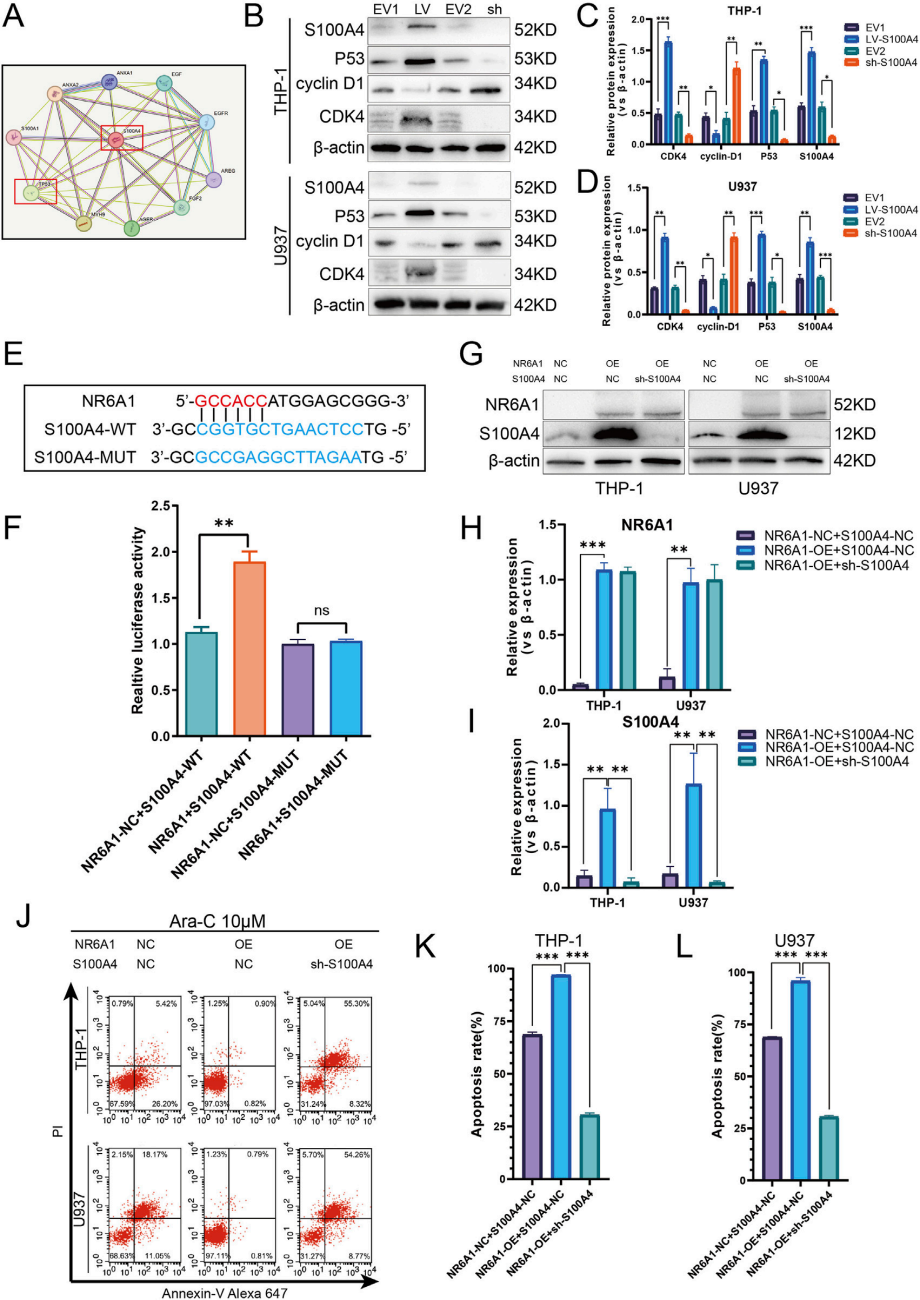
Activation of S100A4 by Transcription Factor NR6A1 and Its Role in Mediating Cytarabine Resistance via the p53 Signaling Pathway. **(A)** Protein-protein interaction network of S100A4 and TP53 (STRING). **(B)** The protein expression levels of S100A4, p53, cyclin D1, CDK4 was evaluated by western blotting analysis in the EV1, LV-S100A4, EV2 and sh-S100A4 groups. **(C, D)** The relative gray values were shown in histogram. **(E)** Dual luciferase assays were performed to detect the binding of the transcription factor NR6A1 to the promoter sequence region of the target gene S100A4 in order to investigate the regulation of the target gene by the transcription factor. **(F)** Luciferase experiments with the wild-type and the mutated 3′ UTR of KIAA1522. **(G)** The protein expression of S100A4 and NR6A1 in the “NR6A1-NC + S100A4-NC”. “NR6A1-Overexpression + S100A4-NC” and “NR6A1- Overexpression + sh-S100A4” groups. **(H, I)** The relative gray values were shown in histogram. **(J, K, L)** After treated with 10 μM Ara-C for 24 h, the percentage of apoptotic cells was demonstrated by flow cytometry in the “NR6A1-NC + S100A4-NC”, “NR6A1-Overexpression + S100A4-NC” and “NR6A1- Overexpression + sh-S100A4” groups. Data are shown as mean ± SD representing three biological replicates. *p < 0.05, **p < 0.01, ***p < 0.001, ns, no significant difference.

In the course of our prior research, we have demonstrated that S100A4 varies between AML cell-sensitive and drug-resistant strains, thereby modifying the capacity of cells to resist Ara-C-induced death. To further explore the mechanism by which S100A4 regulates cellular resistance to Ara-C, we found a close association between S100A4 and TP53, a key cell cycle protein, using protein-protein interaction (PPI) network interaction analysis (Figure 8A). To verify their relationship, we detected the expression of cell cycle key proteins p53, cyclin-D1, and CDK4 after regulating S100A4 expression. The results showed that p53 expression was elevated, cyclin-D1 expression was decreased, and CDK4 expression was elevated in the LV-S100A4 group compared with the EV1 group; p53 expression was decreased, cyclin-D1 expression was elevated, and CDK4 expression was decreased in the sh-S100A4 group compared with the EV2 group (Figures 8B–D). These findings suggest that S100A4 high expression can mediate AML tumor cells undergoing cell cycle arrest by enhancing p53 expression.

In addition, a combination of the UCSC and JASPAR databases was utilized to investigate the upstream transcription factors capable of enhancing S100A4 transcription. The results of dual luciferase assays demonstrated the direct binding of NR6A1 to the S100A4 promoter and the binding sequence between them (Figures 8E,F). Furthermore, Western blot detection of S100A4 expression after overexpression-regulation of NR6A1 expression revealed elevated S100A4 expression (Figures 8G–I). The FCM detection of apoptosis rate after Ara-C stimulation revealed that the NR6A1-OE + S100A4-NC group was smaller than the NR6A1-NC + S100A4-NC group, but the apoptosis rate was higher in the NR6A1-OE + sh-S100A4 group than in the NR6A1-OE + S100A4-NC group (Figures 8J–L).

## Discussion

Despite significant progress in the treatment of AML in recent years (Kantarjian H. M. et al., 2024),drug resistance (DiNardo and Wei, 2020) limited treatment options for elderly patients (Li et al., 2024) the challenge of individualized therapy, and treatment-related complications remain current dilemmas (Kantarjian et al., 2021). Ara-C, a core chemotherapeutic agent for the treatment of AML, is indispensable in the induction, intensification, and consolidation of AML, and is an important component of the standard treatment regimen. The “3 + 7” regimen has been widely used in the treatment of AML over the past few decades, resulting in complete remission in 60%–80% of AML patients and remains one of the most commonly used treatment regimens today (Lancet et al., 2018; Lancet et al., 2021). After AML patients have achieved complete remission, cytarabine is one of the main agents used in consolidation chemotherapy to prevent disease recurrence (Bornhäuser et al., 2023). Cytarabine is one of the mainstays of chemotherapy for the prevention of disease recurrence. Cytarabine is often used in combination with other drugs to improve the therapeutic effect. For example, the combination of cytarabine with gitumumab (GO) has shown synergistic effects in certain subtypes of AML (Borthakur et al., 2022). Despite the development of a variety of new targeted therapeutic agents and immunotherapeutic agents in recent years, the use of these new drugs in the treatment of AML is still limited. The position of cytarabine in AML treatment remains irreplaceable. Despite the importance of cytarabine in AML treatment, the emergence of drug resistance remains a serious problem. Therefore, there is a need to identify novel biomarkers that can predict not only the prognosis of AML, but also the therapeutic response to cytarabine-based therapy. In this study, we analyzed potential mechanisms of cytarabine resistance using bioinformatics tools. The establishment of an ARRGRS consisting of 10 biomarkers (S100A4, ASCC3,EPB41L2,NET1,TEX30,CSPG4, MPO,PDE4A, RASAL3,SHANK1) based on differential genes associated with cytarabine resistance was used to categorize AML patients, which helped to assess the overall survival of these patients and response to cytarabine-based therapy. Both in the TCGA cohort and in the external validation cohort (Beat AML), ARRGRS was able to successfully categorize AML patients into high/low risk groups. The high-risk group had more malignant clinical features and shorter survival times compared to the low-risk group. In addition, we categorized AML patients into two different clusters based on 357 cytarabine resistance-associated DEGs. Cluster 2 had worse clinical features and shorter survival time compared to cluster 1, suggesting that cluster categorization has prognostic and clinical value. The characterization of cytarabine resistance-related genes will help to reveal the mechanism of AML cytarabine resistance and provide insights for optimizing the treatment of AML patients. To explore the potential biological functions of the high and low ARRGRS subgroups, we performed GESA and GO/KEGG analyses. Cell adhesion, chemokine signaling pathways, and cytokine-cytokine receptor interaction-related pathways were mainly enriched in the high ARRGRS group. The chemokine signaling pathway has multiple important functions in AML, e.g., increased secretion of the chemokine target CXCL10 in the bone marrow microenvironment may affect clonal selection and disease evolution (Chen et al., 2024). Chemokines are not only involved in the construction of the bone marrow microenvironment, but also play an important role in the proliferation and apoptosis of AML cells and in the modulation of immunity (Masamoto et al., 2023).

**FIGURE 9:**
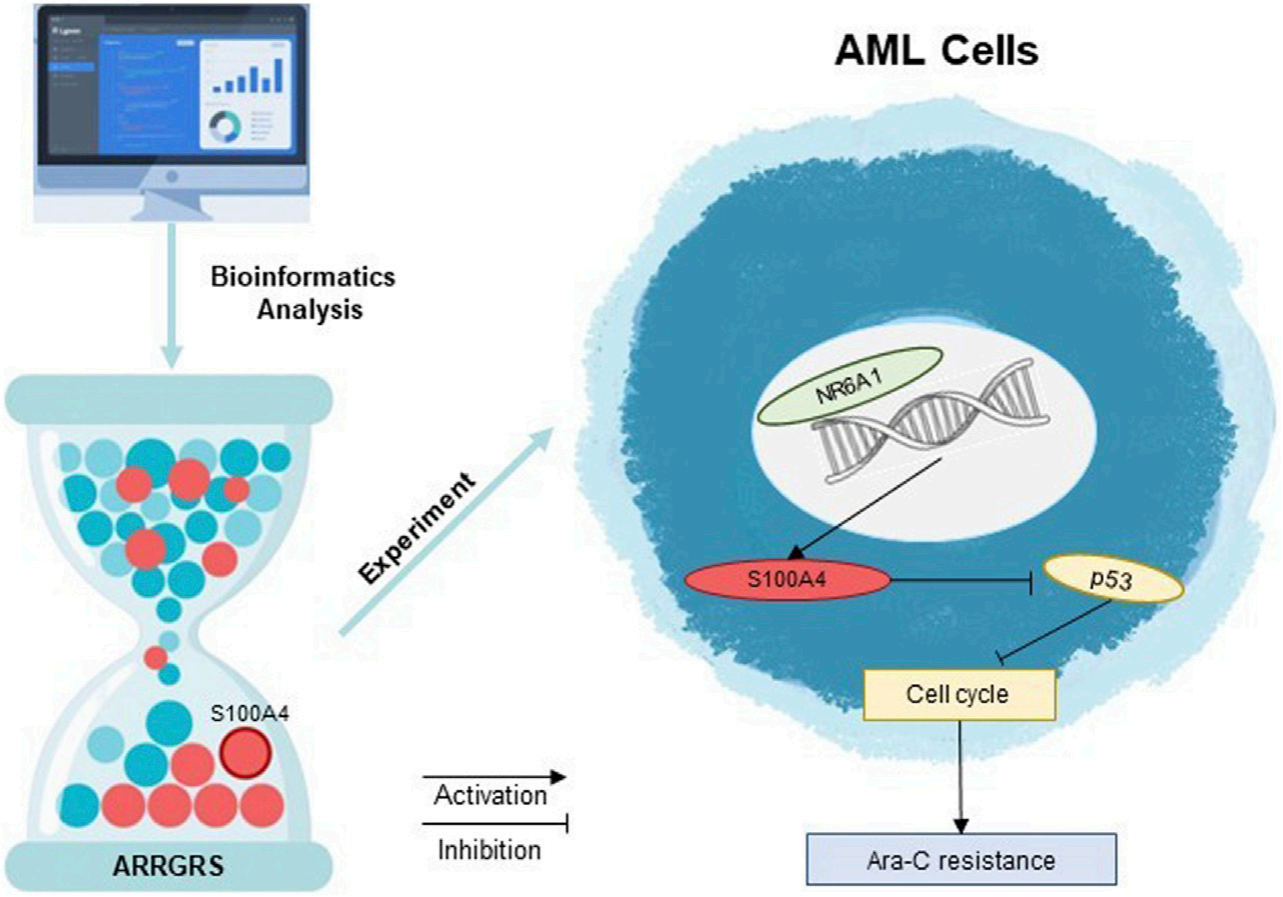
Decoding Ara-C resistance through ARRGRS: The oncogenic circuitry of NR6A1-Driven S100A4/p53 activation in AML.

In this study, we found that these 10 cytarabine-associated genes were differentially expressed in both normal and AML patient samples. The results of survival analysis suggested that S100A4, SHANK1, MPO might be more clinically relevant. Interestingly, SHANK1, MPO expression was higher in AML patients, but survival analysis showed better survival outcomes in the SHANK1, MPO high expression group. In order to select the final 10 genes to compare the expression differences between AML arabinoside-resistant and sensitive strains by experiments, and to synthesize the results of the biosignature expression analysis, survival analysis and experimental analysis, we firstly chose S100A4 to investigate its role in arabinoside resistance. There is increasing evidence that S100A4 overexpression in AML is a poor prognostic biomarker with the potential to guide clinical treatment planning (Alanazi et al., 2020; Yao et al., 2023). However, most studies on S100A4 in AML are based on algorithms of raw-fiducial analysis, and studies on the molecular mechanisms of the roles of S100A4 are lacking. In this study, S100A4 was detected to be expressed at much higher levels in cytarabine-resistant cell lines than in sensitive lines. The results of the next experiments indicated that upregulation of S100A4 expression in THP-1, U937 cell line could increase the cellular tolerance to cytarabine, while downregulation of S100A4 expression could enhance the sensitivity of THP-1, U937 to cytarabine. The results of *in vivo* experiments similarly indicated that downregulation of S100A4 expression could increase the sensitivity of tumor cells to cytarabine and inhibit tumor growth. This study revealed for the first time the relationship between the S100A4 protein and AML cytarabine resistance. The PPI protein interaction network indicated a close association between S100A4 and TP53 (Szklarczyk et al., 2023). Several studies have demonstrated that TP53 plays an important role in participating in the regulation of AML drug resistance (DiNardo et al., 2020b; Nechiporuk et al., 2019). The P53 protein encoded by the TP53 gene, an important tumor suppressor protein, is important in the cell cycle regulation, DNA damage repair, and apoptosis (Zawacka, 2024; Man et al., 2023). Ara-C plays an anticancer role by inhibiting cell proliferation mainly through interfering with DNA synthesis. Specifically, Ara-C acts mainly in the S phase of the cell cycle, preventing DNA strand elongation by inhibiting the activity of DNA polymerase (Yamauchi et al., 2009). AML cells may develop drug resistance after prolonged exposure to Ara-C, which has been linked to cell cycle regulation (Ling et al., 2023; Matthews et al., 2001). In addition, some studies have found that drug-resistant AML cells may be able to inhibit the proliferation of cells through the upregulation of certain genes associated with cell cycle arrest genes, such as TP53, to resist the induction of apoptosis by Ara-C (DiNardo et al., 2020a; Grob et al., 2022). Therefore, we explored the relationship between S100A4 and the p53 signaling pathway, as well as cyclin D1, a key protein in cell cycle checkpoints. It was found that p53 expression was elevated when S100A4 expression was enhanced, cyclin D1 was inhibited, and synthesis of the cyclin/CDK complex was blocked suggesting that AML tumor cells are blocked from entering S phase in G1 phase. Conversely, knockdown of S100A4 decreased p53 expression and elevated cyclin D1 expression. To further investigate the reason for the high expression of S100A4 in cytarabine-resistant AML cells, we found that NR6A1 could directly bind to the S100A4 promoter region to achieve enhanced transcription by analyzing multiple transcription factors. In addition, our results demonstrated that the increase in cellular drug resistance mediated by high NR6A1 expression was mainly achieved by upregulating the S100A4 expression level, in other words, even though NR6A1 expression was elevated when the S100A4 expression level was knocked down, it did not increase the resistance of AML cells to cytarabine. This means that the expression of NR6A1 can be reduced to achieve the purpose of decreasing the expression level of S100A4 in cytarabine-resistant AML cells. From the above results, it is suggested that targeting S100A4 may be a useful approach to reverse cytarabine resistance (Figure 9).

Our study has several limitations. First, the prognostic significance of the ARRGRS model was only validated using public cohorts from the TCGA or Beat AML databases, and more clinical cohorts, including chemotherapy/immunotherapy cohorts, should be included to confirm our findings. Second, we validated the role of S100A4 in cytarabine-resistant cell lines only *in vitro* and *in vivo*, and further exploration of the expression profile of S100A4 in patients with AML who are not responding favorably to cytarabine-based therapies will elucidate the clinical value of S100A4. In this study, the ARRGRS model was developed to help predict overall survival and chemotherapy response in AML patients. Third, the present study has not explored the interaction between the ARRGRS scoring system and the immune microenvironment, and the analysis of the related mechanisms will be reported as a separate research direction. In the future, it would be interesting to conduct clinical trials to explore whether the ARRGRS model can predict response to chemo-immunotherapy. Although the involvement of S100A4 in cytarabine resistance has also been preliminarily validated, revealing the mechanism of S100A4 in chemotherapy resistance, such as the effects on DNA damage repair, drug metabolism, and tumor microenvironment, would be helpful to develop some drugs targeting S100A4 to inhibit tumor growth and overcome chemotherapy resistance. Besides, it is also interesting to explore the relationship between S100A4 and other chemotherapeutic agents such as gemcitabine, flexibiotics, and vincristine, which may broaden the clinical applications of S100A4 knockdown, such as the treatment of other cancers and cytarabine high-dose therapy.

## Data Availability

The raw data supporting the conclusions of this article will be made available by the authors, without undue reservation.

## Glossary

AML: Acute Myeloid Leukemia
Ara-C: cytarabine
ARRGRS: Ara-C resistance-related gene risk score
AUC: Area Under Curve
CCK8: Cell counting Kit-8 assay
CDK: cyclin-dependent kinases
DEGs: Differentially expressed genes
DNA: DeoxyriboNucleic Acid
EDU: 5-ethynyl-2′-deoxyuridine
EV1: Empty virus of LV
EV2: Empty virus of RNAi
FCM: Flow Cytometry
GEO: Gene Expression Omnibus
GO: Gene Ontology
GSEA: Gene Set EnrichmentAnalysis
GTEx: Genotype-Tissue Expression
IHC: Immunohistochemical
KEGG: Kyoto Encyclopedia of Genes and Genomes
K-M: Kaplan-Meier
NC: Negative Control
NES: Normalize enrichment score
NR6A1: Nuclear Receptor Subfamily 6 Group A Member 1
OE: Overexpression
OS: Overall Survival
PPI: Protein-Protein Interaction Networks
RNA: RibonucleicAcid
ROC: Receiver Operating characteristic
RT-PCR: Real time-PCR; S100A4
S100: Calcium Binding Protein A4
TCGA: The Cancer Genome Atlas
UCSC: University of California Santa Cruz
