# Supplementary material for: Identification of an Ara-C resistance-related gene risk score and the role of S100A4 in AML via NR6A1-dependent activation and p53 regulation
Source: Front Pharmacol. 2025 Jun 13;16:1574759. doi: 10.3389/fphar.2025.1574759 (PMC12202665; doi:10.3389/fphar.2025.1574759)
Supplement: Supplementary file 1 [file DataSheet1.zip › Supplementary/Supplementary Figure1.docx]

| Supplementary Table 1 Primer sequences used for qRT-PCR | | | |
| --- | --- | --- | --- |
| Primer name |  |  | Sequence（5’-3'） |
| S100A4-F |  |  | GAGCAACTTGGACAGCAACAG |
| S100A4-R |  |  | CCCAACCACATCAGAGGAGT |
| ASCC3-F |  |  | CGTACTTTACGGCAGGTGGA |
| ASCC3-R |  |  | AGGTACTGGTCGAAAACGGC |
| EPB41L2-F |  |  | TGCCAACACCAAACCTTTCC |
| EPB41L2-R |  |  | CTTGGGCTGGCTTTGTTCAC |
| NET1-F |  |  | TCAGACGGCAGGAGGCAATA |
| NET1-R |  |  | GCTCCACTGTTCCATCAGGC |
| TEX30-F |  |  | GCAGAGCAGCGTTATCTTCA |
| TEX30-R |  |  | CTTCAGGGTCCCCATATTTGA |
| CSPG4-F |  |  | TATGGGCTCTTTGTTGGGGG |
| CSPG4-R |  |  | GGGTTCCTTCGTCCTGAGTG |
| MPO-F |  |  | TTTTGGTGGGAGAACGAGGG |
| MPO-R |  |  | AAGCCAGGTTCAATGCAGGA |
| PDE4A-F |  |  | TCACCCACGATGAAGGAACG |
| PDE4A-R |  |  | TCGGTCTTCACCCCAAATCG |
| RASAL3-F |  |  | TCCGGGAAAACACATTGGC |
| RASAL3-R |  |  | GCTCCGAGGCTGGACATTTG |
| SHANK1-F |  |  | ACGTGCGGTTCCTTGAAAAC |
| SHANK1-R |  |  | AGCGGTTCCACGAAAAGGAA |
| β-actin-F |  |  | CTCGCCTTTGCCGATCC |
| β-actin-R |  |  | ATCCTTCTGACCCATGCCC |


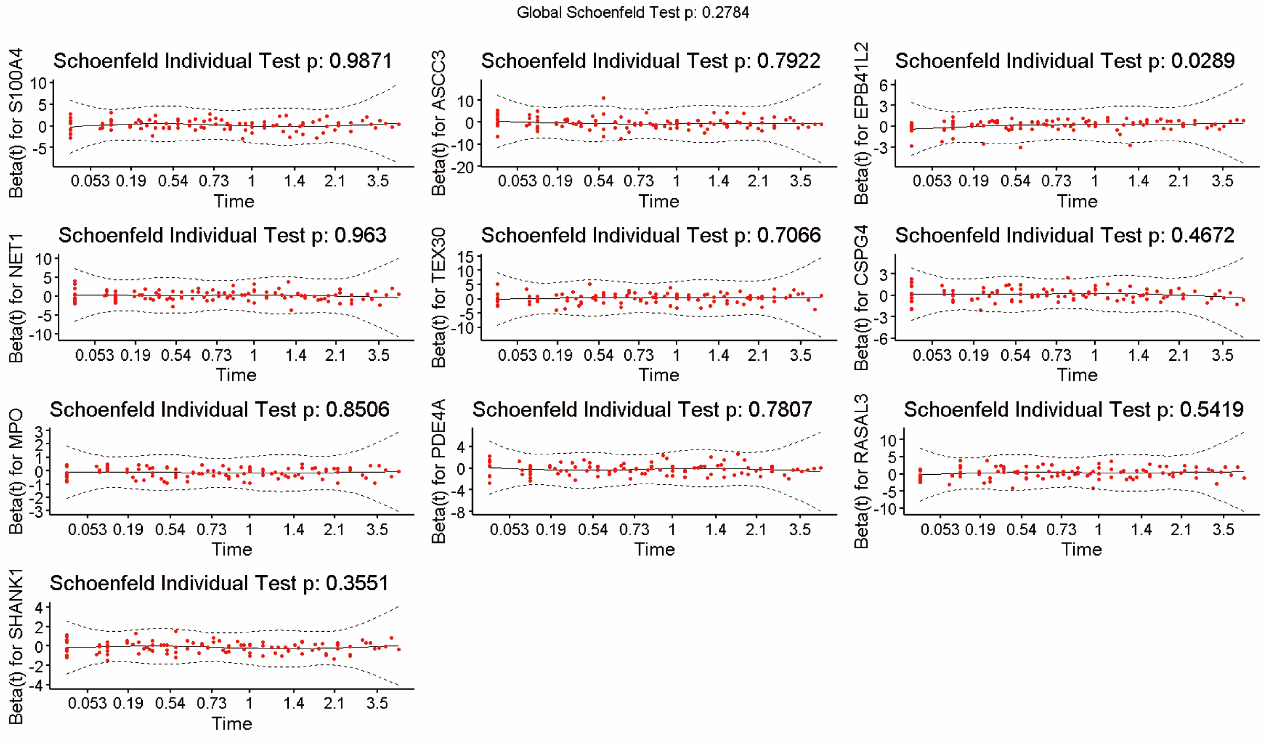


Supplementary Figure 1 Schoenfeld residual test results for 10 genes in the ARRGRS score. Each subplot shows the residuals for different genes over time (red points are residual values, black solid lines are smoothed curves, and dashed lines are 95% confidence intervals), with corresponding Schoenfeld individual test p-values labeled, and the global Schoenfeld test p-value (0.2784) is given in the upper corner.
