# Supplementary figures and images for: Identification of an Ara-C resistance-related gene risk score and the role of S100A4 in AML via NR6A1-dependent activation and p53 regulation
Source: Front Pharmacol. 2025 Jun 13;16:1574759. doi: 10.3389/fphar.2025.1574759 (PMC12202665; doi:10.3389/fphar.2025.1574759)

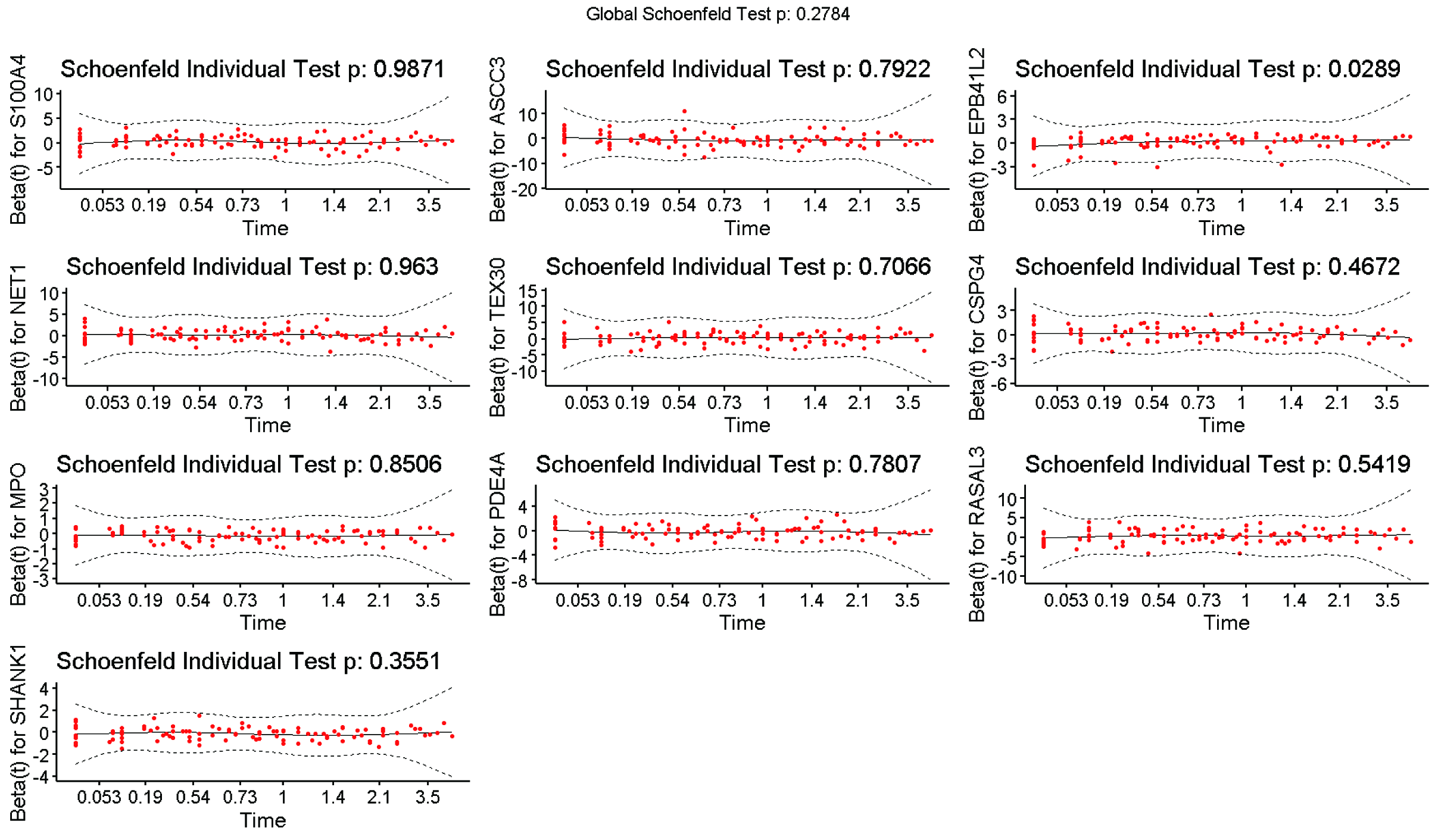

Supplement: Supplementary file 1 [file DataSheet1.zip › Supplementary/S1.tif]
